# Identification of Endoplasmic reticulum Homeostasis-associated diagnostic biomarkers for Sarcopenia through integrated Bioinformatics and Experimental Validation

**DOI:** 10.7717/peerj.21903

**Published:** 2026-11-02

**Authors:** Long Shi, Hongyu Yan, Junliang Lu, Wenkai Yu, Yang Li, Zhiwen Zhang

**Affiliations:** 1 School of Acupuncture and Orthopedics, Hubei University of Traditional Chinese Medicine, Wuhan, China; 2 The Third Affiliated Hospital of Beijing University of Chinese Medicine, Beijing, China; 3 First Clinical Medical College, Hubei University of Traditional Chinese Medicine, Wuhan, China; 4 State Key Laboratory of Magnetic Resonance and Imaging, National Centre for Magnetic Resonance in Wuhan, Innovation Academy of Precision Measurement Science and Technology, Chinese Academy of Sciences (CAS), Wuhan, China; 5 Department of Orthopedic Surgery, Hubei Provincial Hospital of Traditional Chinese Medicine /Affiliated Hospital of Hubei University of Chinese Medicine, Wuhan, China; 6 Hubei Key Laboratory of Theory and Application Research of Liver and Kidney in Traditional Chinese Medicine (Hubei Province Hospital of Traditional Chinese Medicine), Wuhan, China; 7 Hubei Shizhen Laboratory, Wuhan, China

**Keywords:** Sarcopenia, Function information, Diagnostic value, ER-stress

## Abstract

Sarcopenia is an age-related skeletal muscle disorder characterized by progressive loss of muscle mass and function, yet reliable biomarkers for early diagnosis remain limited. Emerging evidence suggests that disruption of endoplasmic reticulum (ER) homeostasis contributes to skeletal muscle aging, however, the diagnostic relevance of ER stress- and reticulophagy-related alterations in sarcopenia remains poorly understood. In this study, three skeletal muscle transcriptomic datasets (GSE25941, GSE28422, and GSE38718) were integrated following batch-effect correction to identify differentially expressed genes (DEGs) between sarcopenic and control samples. DEGs were intersected with ER stress and reticulophagy related gene sets to obtain sarcopenia-associated candidate genes. Subsequently, three machine-learning algorithms, including least absolute shrinkage and selection operator (LASSO), random forest (RF), and support vector machine (SVM), were applied to identify key hub genes. Functional enrichment analyses, transcription factor (TF)-miRNA regulatory network construction, protein-chemical interaction analyses, external validation, nomogram development, and quantitative real-time PCR (qRT-PCR) verification were performed. A total of 236 DEGs were identified, comprising 34 upregulated and 202 downregulated genes. Intersection analysis with 1,772 ER stress and reticulophagy related genes yielded 10 candidate genes, from which five hub genes (*ACTC1*, *NPY*, *CDKN2B*, *GADD45G*, and *GRIA1*) were consistently identified by machine-learning analyses. Functional enrichment analysis indicated significant involvement in extracellular matrix organization, cytokine-cytokine receptor interaction, mitochondrial translation, and respiratory electron transport pathways. Regulatory network analyses further revealed potential interactions with multiple transcription factors, miRNAs, and chemical compounds. In the independent validation cohort (GSE1428), *ACTC1* showed strong individual discriminative performance, *NPY* and* GADD45G* showed moderate performance, whereas *GRIA1* showed limited discrimination and *CDKN2B* showed near-chance individual performance, whereas the combined gene signature exhibited improved discriminative ability. A predictive nomogram was established. Moreover, qRT-PCR analysis of human skeletal muscle biopsy specimens confirmed significantly increased expression of *ACTC1*,* NPY*,* CDKN2B*, and* GRIA1*, together with decreased expression of *GADD45G* in sarcopenic muscle tissues compared with controls (all *p* < 0.05). Collectively, our findings identified a five-gene signature associated with ER stress and reticulophagy related molecular networks in sarcopenia. Although these genes are not established core regulators of ER stress or reticulophagy, they exhibited promising diagnostic value and may reflect ER homeostasis-related molecular alterations during skeletal muscle aging, providing novel biomarkers and potential insights into the molecular basis of sarcopenia.

## Introduction

Sarcopenia is defined as the progressive, age-related loss of skeletal muscle mass and function. It constitutes a substantial public health burden, markedly increasing the risk of falls, frailty and premature death in older populations (Cruz-Jentoft & Sayer, 2019). It is estimated that sarcopenia affects 10–16% of the elderly population globally (Petermann-Rocha et al., 2022). However, the field is currently limited by diagnostic approaches that primarily identify muscle loss after clinically appreciable deterioration has occurred, such as assessments of muscle mass, grip strength, and gait speed (Coletta & Phillips, 2023; Ooi & Welch, 2024). It is also constrained by the lack of widely approved disease-modifying pharmacological therapies specifically indicated for sarcopenia (Bahat & Ozkok, 2024). The urgent and pressing priority of current research is to fully elucidate the molecular mechanisms of sarcopenia and identify specific biomarkers. The pathophysiology of sarcopenia defies simple explanation. Hormonal decline, chronic inflammation, and physical inactivity have been identified as contributing factors (Shur et al., 2021; Marmondi et al., 2025). However, these factors alone do not fully account for the precipitous atrophy observed in aged muscle. A mounting body of evidence has emerged that identifies the disruption of intracellular proteostasis as a convergent mechanism (Gallot & Bohnert, 2021).

The endoplasmic reticulum (ER) is the main organelle responsible for protein folding and calcium homeostasis. Aging disrupts ER homeostasis by reducing the efficiency of protein quality-control systems, thereby promoting the accumulation of aberrant proteins and chronic activation of ER stress responses (Bohnert, McMillan & Kumar, 2018; Paez, Pitzer & Alway, 2023). This stress exerts a bidirectional destructive effect on the body. On the one hand, it activates catabolic signals and apoptosis pathways, accelerating muscle fiber loss. On the other hand, it induces synthetic metabolic resistance, a persistent state in which aged muscle cannot produce a protein synthesis response to ingested amino acids (Zhang et al., 2018; Tang et al., 2019; Wang et al., 2020). Compounding this insult, age-associated mitochondrial dysfunction establishes positive-feedback loops that amplify ER stress, perpetuating a self-sustaining cycle of organelle failure and proteolytic activation (Aragon, Tipton & Schoenfeld, 2023). Although increasing evidence supports a role for ER stress in skeletal muscle aging, the regulatory mechanisms that maintain ER homeostasis under chronic stress conditions remain poorly characterized.

Among these mechanisms, reticulophagy (ER-phagy), a selective form of autophagy that mediates the degradation of damaged or excess ER fragments, has emerged as a critical pathway for preserving ER integrity and proteostasis. By eliminating dysfunctional ER components, reticulophagy helps alleviate excessive ER stress and restore cellular homeostasis, however, its contribution to skeletal muscle aging and sarcopenia remains largely unexplored (Chino & Mizushima, 2023; Wilson & McCormick, 2025).

Current diagnostic practice offers no window into these subcellular events. High-throughput transcriptomics, coupled with machine-learning-based feature selection, now provide a means to identify molecular signatures of ER dysfunction that precede clinical manifestation (Ran et al., 2025; Deng et al., 2026). In this study, we integrated three independent transcriptomic datasets, and intersected differentially expressed genes with a curated compendium of ER-related genes and applied multiple machine-learning algorithms for robust feature selection. The resulting composite model was externally validated and functionally interrogated through enrichment analysis and regulatory network construction, with experimental corroboration by quantitative real-time PCR. These findings deepen our understanding of the molecular mechanisms of sarcopenia and point to potential biomarkers for its diagnosis and management.

## Materials & Methods

### Data standardization and preprocessing

Gene expression datasets were retrieved from the gene expression omnibus (GEO) (http://www.ncbi.nlm.nih.gov/geo/) database using the keyword “skeletal muscle”, “muscle aging”, “aged”, “elderly”, and “sarcopenia”. Datasets were subsequently screened manually according to the following criteria: (1) human skeletal muscle tissue samples; (2) inclusion of aged, elderly, or sarcopenic subjects and healthy controls; (3) available gene expression matrices; and (4) sufficient sample information.

Following manual screening, GSE38718 (Liu et al., 2013), GSE25941, and GSE28422 (Raue et al., 2012) were selected as training datasets, while GSE1428 (Giresi et al., 2005) was used as an external validation dataset. Two researchers independently screened and cross-checked all retrieved datasets. The characteristics of the datasets are detailed in Table 1.

**Table 1:** Detailed information of the selected GEO datasets.

	Dataset	Control	Sarcopenia	Platform
Training set	GSE38718	14	8	GPL570
	GSE25941	15	21	GPL570
	GSE28422	15	12	GPL570
Validation set	GSE1428	10	12	GPL96

Because the public cohorts were not uniformly classified using contemporary Asian working group for sarcopenia (AWGS) or European working group on sarcopenia in older people (EWGSOP) diagnostic criteria, the case labels followed the phenotype definitions provided in the original studies. Where formal clinical sarcopenia criteria were unavailable, the case group should be interpreted as representing an aging-related muscle loss or sarcopenia-related phenotype rather than clinically adjudicated sarcopenia.

All datasets were preprocessed and normalized before analysis. We merged GSE38718, GSE25941, and GSE28422 with the R package “inSilicoMerging” (v1.2.0) (Taminau et al., 2012) and adjusted for batch effects using ComBat from the “sva” package (v3.50.0) (Johnson, Li & Rabinovic, 2007). The resulting training set included 44 control samples and 41 samples with sarcopenia-related or aging-related muscle phenotypes, while GSE1428 was held out as the validation set (10 control samples and 12 phenotype samples). From the GeneCards database (https://www.genecards.org/), we retrieved 12,948 protein-coding genes linked to “ER-Stress” and “reticulophagy”. Following removal of duplicate genes, only genes with a Relevance Score > 5 were retained to prioritize genes with stronger database-supported associations and reduce low-confidence entries, resulting in a final set of 1,772 genes. The gene list is provided in Table S1.

### Identification of differentially expressed genes (DEGs)

Transcriptomic differences between control and sarcopenia groups in the training set were quantified using the “limma” package (v3.52.2) (Ritchie et al., 2015). Differentially expressed genes (DEGs) were defined by the criteria: —log_2_FC— > 0.58 and *p.adj < 0.05*. Resulting DEGs were visualized as volcano plots and clustered heatmaps using ggplot2 (v3.4.4) (Gustavsson et al., 2022) and ComplexHeatmap package (v2.13.1) (Gu, 2022). The gene list is provided in Table S2.

### Gene set enrichment analysis (GSEA)

We conducted GSEA (Subramanian et al., 2005) with “clusterProfiler” R package (v4.10.0) (Wu et al., 2021) to identify enriched pathways between groups, employing two gene set collections. The first was the MSigDB gene set “c2.cp.all.v2022.1.Hs.symbols.gmt” (https://www.gsea-msigdb.org/gsea/msigdb/collections.jsp). The second was ER stress-related bespoke custom gene set, created by filtering MSigDB annotations for “ER-Stress” and “Endoplasmic Reticulum” and consolidating 11 relevant gene sets: (BIOCARTA_BCR_PATHWAY.v2026.1.Hs,

BIOCARTA_EIF2_PATHWAY.v2026.1.Hs,

BIOCARTA_NO1_PATHWAY.v2026.1.Hs,

BIOCARTA_RAB_PATHWAY.v2026.1.Hs,

BOCHKIS_FOXA2_TARGETS.v2026.1.Hs,

BUYTAERT_PHOTODYNAMIC_THERAPY_STRESS_DN.v2026.1.Hs,

BUYTAERT_PHOTODYNAMIC_THERAPY_STRESS_UP.v2026.1.Hs,

FAN_OVARY_CL0_XBP1_SELK_HIGH_STROMAL_CELL.v2026.1.Hs,

FAN_OVARY_CL1_GPRC5A_TNFRS12A_HIGH_SELECTABLE_FOLLICLE_ STROMAL_CELL.v2026.1.Hs,

GOBP_ATF6_MEDIATED_UNFOLDED_PROTEIN_RESPONSE.v2026.1.Hs,

REACTOME_NFE2L2_REGULATING_ER_STRESS_ASSOCIATED_ GENES.v2026.1.Hs).

Input comprised a ranked gene list sorted by signed differential expression log_2_FC. Enrichment significance required p.adj < 0.05, FDR < 0.05, and —NES— > 1.5. Top-ranking pathways were plotted with ggplot2 (v3.4.4).

### Gene set variation analysis (GSVA)

The assessment of single-sample pathway enrichment was facilitated by the implementation of GSVA (Hänzelmann, Castelo & Guinney, 2013) within the GSVA package (v1.50.0), thereby enabling the identification of sample-specific activity alterations associated with sarcopenia. The reference collection, designated c2.cp.v7.4.symbols.gmt, was retrieved from MSigDB (accessed 10 May 2026). The classification of a pathway as significantly altered was determined by the fulfilment of the following criteria: the *—t-score—* must exceed 4 and the p.adj < 0.05. The resulting top 10 pathways, both positively and negatively enriched, were then plotted in the form of a bar chart.

### Identification and functional enrichment analysis of Sa-ER RGs

A Venn overlap analysis was performed using the VennDiagram package (version 1.7.3) (Chen & Boutros, 2011) to identify genes that were present in the DEGs, ER stress and reticulophagy related gene. These intersecting genes were labelled “Sarcopenia-Endoplasmic Reticulum stress and reticulophagy related Genes (Sa-ER RGs)”. To optimise candidate selection and guard against elevated false discovery rates, we assessed their expression in the training cohort using Wilcoxon test (Li et al., 2022).

Functional and pathway enrichment analyses were performed with clusterProfiler (v4.10.0), covering GO and KEGG categories. Significance was set at adjusted *p* < 0.05.

### Screening for hub genes using machine learning

In order to distil a robust diagnostic gene signature for sarcopenia, three distinct machine learning strategies were integrated. Least absolute shrinkage and selection operator (LASSO) regression (glmnet v4.1.10) (Engebretsen & Bohlin, 2019) was applied for penalized feature selection under 10-fold cross-validation, with the objective of optimising both predictive performance and interpretability. The random forest (RF) (“randomForest” v4.7.1.2) (Wang et al., 2023) ensemble classifier was deployed in order to circumvent the overfitting of decision trees and enhance the generalisability of the results. Support vector machine (SVM) (“e1071” v1.7.16) was employed with 10-fold cross-validation, capitalising on its suitability for high-dimensional, non-linear classification tasks. Hub genes that achieved consensus across all three analytical frameworks were retained as high-confidence diagnostic candidates. The chromosomal architecture of these samples was subsequently visualised using “Circlize” R package (v0.4.16) (Gu et al., 2014).

### TF-miRNA and hub gene interactome construction

To analyse the regulatory landscape upstream of the core genes, we used NetworkAnalyst (https://www.networkanalyst.ca/), to infer their putative transcription factors (TFs) and microRNAs (miRNAs). We visualised the resulting TF–miRNA–gene regulatory and protein–chemical interaction networks in Cytoscape (v3.10.4). The CytoNCA plugin (Tang et al., 2015) was used to quantify degree centrality (DC), betweenness centrality (BC), closeness centrality (CC), and eigenvector centrality (EC). All nodes in the TF-miRNA-gene network were included in a single topological screening step. Core nodes were defined using the criteria Degree > 2 and BC, CC, and EC values exceeding their respective medians; the Degree > 2 threshold was used to exclude nodes with limited network connectivity.

### Construction of a gene-gene interaction network of hub genes

To map the functional interactome of the hub genes, we used GeneMANIA (Franz et al., 2018) (https://genemania.org/, accessed 15 May 2026) to identify biologically relevant network partners. This platform uses multi-omics evidence to extend query-based networks, enabling the construction of an expanded gene interaction map for functional interpretation.

### Construction and validation of a diagnostic model

The diagnostic efficacy of the hub genes was evaluated at three levels: single-gene performance, composite model construction and independent validation in GSE1428. Discrimination was quantified by the area under the curve (AUC) (95% confidence interval (CI)) from receiver operating characteristic (ROC) analysis (“pROC” v1.18.0) (Robin et al., 2011), with visualisation using the “ggplot2” package. An AUC greater than 0.7 was considered clinically relevant (Mandrekar, 2010). Calibration was examined through predicted *versus* observed probability plots (“rms” v6.4.0; “ResourceSelection” v0.3-5), and net benefit was examined through DCA (“rmda” v1.6). A clinical nomogram was also developed (“rms” v6.4.0).

### Clinical samples collection

Vastus lateralis muscle specimens were collected from patients undergoing hip surgery. The sarcopenia group consisted of 6 patients (mean age, 75.3 ± 11.6 years), whereas the non-sarcopenic control group included 6 patients (mean age, 43.7  ± 12.5 years). Diagnostic classification followed the 2019 AWGS guidelines (Chen et al., 2020). Tissue processing occurred within 15 min of excision: formalin fixation (4%) for histopathology and cryopreservation in liquid nitrogen at −80 °C for molecular analyses. The Hubei Provincial Hospital of Traditional Chinese Medicine provided ethical oversight (approval HBZY-2024-C102-02, dated January 23, 2025), and all participants provided written informed consent.

### Quantitative Real-Time PCR (qRT-PCR) analysis of hub genes

The RNA was purified from frozen muscle samples using RNA extraction kit (TSP413, Tsingke Biotech, Beijing, China), with quality control by micro-spectrophotometry. The reverse transcription process was carried out utilizing RNA using the SynScript^®^ III RT SuperMix for qPCR kit, while the quantitative polymerase chain reaction (qPCR) amplification was conducted employing ArtiCanATM SYBR qPCR Mix kit (TSE501, Tsingke Biotech, China). GAPDH was employed as the reference gene, and relative expression levels were determined by the 2^−ΔΔCT^ method. The primer sequences are presented in Table 2. The raw data can be found in Table S3.

**Table 2:** Primer sequences of target genes.

Gene	Sequence (5′–3′)	bp
NPY	F: TGTTCCCAGAACTCGGCTTG	129 bp
	R: TGCATTGGTAGGATGGGTGG	
CDKN2B	F: AGAAGGACGACGGGAGGGTAATG	122 bp
	R: GATAATCCACCGTTGGCCGTAAA	
GADD45G	F: ACACAGTTCCGGAAAGCACA	116 bp
	R: TTTGGCTGACTCGTAGACGC	
GRIA1	F: AGTTGGTGTTAACGCTGCAG	88 bp
	R: TTGCTTGCTTGCTTCCTTCC	
ACTC1	F: CCTCCCCTCCTCAACCTG	189 bp
	R: ATACCCACCATAACTCCCTGG	
GAPDH	F: AATGGGCAGCCGTTAGGAAA	166 bp
	R: GCCCAATACGACCAAATCAGAG	

### Statistical analysis

Statistical analyses were performed using R (version 4.4.2). Two-tailed t-tests and Wilcoxon rank-sum tests were used for two-group comparisons, depending on the distribution of the data. One-way analysis of variance (ANOVA) with Benjamini–Hochberg correction was applied to multi-group analyses. Pearson’s correlation coefficients were used to assess linear relationships between continuous variables. Statistical significance was defined as *p* < 0.05.

## Results

### Data standardization and preprocessing

The workflow of this study was shown in Fig. 1.

**Figure 1:**
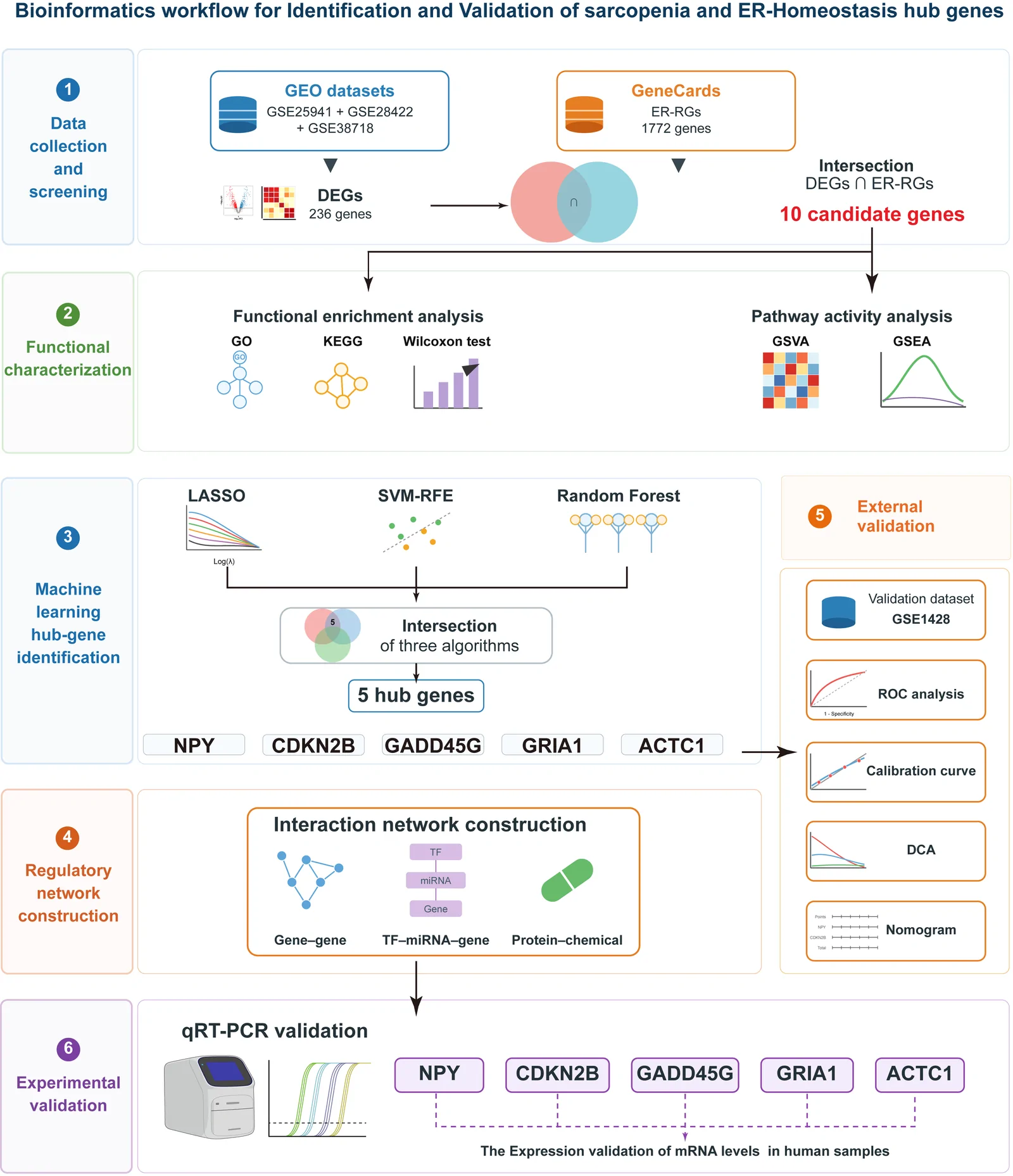
The flowchart of the overall study design.

Preliminary quality control revealed heterogeneity among different independent cohorts in the three publicly available skeletal muscle microarray datasets (GSE25941, GSE28422, and GSE38718) in terms of gene coverage and expression dynamics (Fig. 2A).

**Figure 2:**
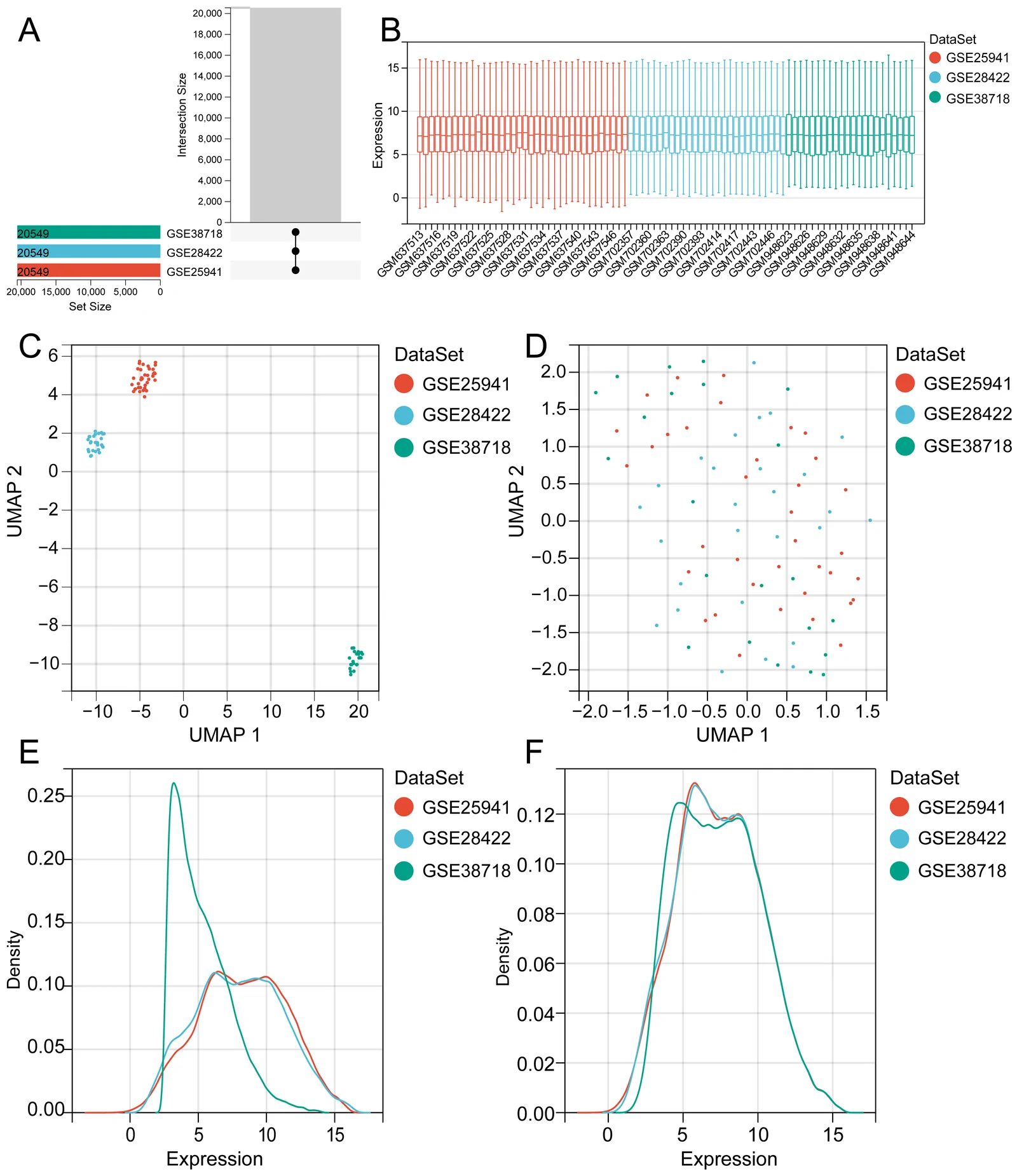
Data standardization. (A) Upset plot illustrating the intersection of genes profiled across the three datasets (GSE25941, GSE28422, and GSE38718). (B) Box plot of gene expression distributions after batch correction. (C–D) UMAP visualization of samples before and after batch correction. (E–F) Density plots of expression distributions before and after batch correction.

Following batch correction, the expression distributions of the three datasets were aligned, with the medians falling on the same level (Fig. 2B). Uniform manifold approximation and projection (UMAP) visualization demonstrated clear dataset-specific clustering before batch correction (Fig. 2C). After correction, samples from the three datasets were intermingled, with no dataset-driven separation observed, indicating that batch effects were effectively eliminated (Fig. 2D). Density plots showed distinct expression distributions before correction (Fig. 2E), whereas the distributions became comparable after correction, with similar means and variances (Fig. 2F).

### Gene set enrichment and variation analysis of the training set

GSEA identified three positively enriched pathways: Classic Pathway (NES = 2.128), ECM Glycoproteins (NES = 1.968), and Core Matrisome (NES = 1.962) (Figs. 3A–3C). Three pathways were negatively enriched: Respiratory Electron Transport (NES = −2.609), Mitochondrial Translation (NES = −2.601), and the Citric Acid TCA Cycle and Respiratory Electron Transport (NES = −2.571) (Figs. 3D–3F, Table S4). GSVA revealed that pathways with positive t-scores included metabolic reprogramming in colon cancer, Parkinson’s disease, Huntington’s disease, mitochondrial translation, the electron transport chain, the oxidative phosphorylation system, complex I biogenesis, nonalcoholic fatty liver disease, mitochondrial calcium uptake, the mitochondrial complex I assembly model. Pathways with negative t-scores included FGFR2 ligand binding and activation, C3 and C5 activation, Runx3-regulated CDKN1A transcription, phase 1 fast channel inactivation, zinc homeostasis, complement activation, and the metabolism of ingested SeMet Sec Mesic into H2Se (Fig. 3G). Custom gene set enrichment analysis revealed negative enrichment trends for photodynamic therapy stress response, RAB pathway, and B-cell receptor signaling pathway, whereas EIF2 signaling pathway showed weak positive enrichment. None of these four custom gene sets reached statistical significance (Fig. S1).

**Figure 3:**
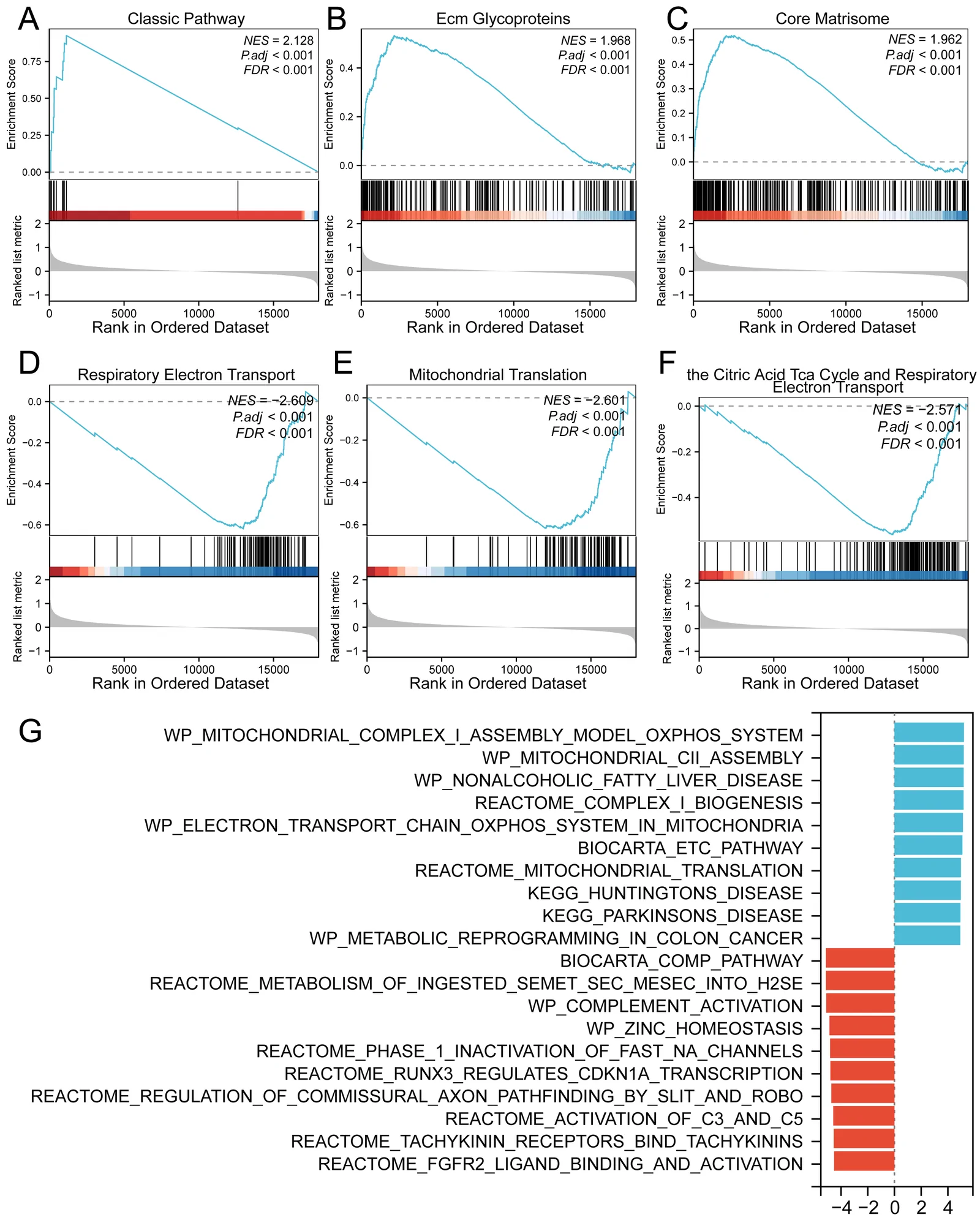
Gene set enrichment and variation analysis of the training set. (A–C) GSEA plots showing positively enriched gene sets. (D–F) GSEA plots showing negatively enriched gene sets. (G) GSVA bar plot ranking pathways by t score. The red bar chart shows the pathways that were significantly upregulated in the sarcopenia group. The blue bar chart shows the pathways that were significantly downregulated in the sarcopenia group.

### Identification and functional characterization of Sa-ER RGs

A total of 236 DEGs were identified, comprising 34 upregulated and 202 downregulated genes (Fig. 4A). The expression patterns of the top 25 DEGs in each category are displayed in a heatmap (Fig. 4B). Cross-referencing the 236 DEGs with 1,772 ER stress- and reticulophagy-related genes identified 10 Sa-ER RGs (Fig. 4C). All Sa-ER RGs showed significant differences between the control and sarcopenia groups (*p* < 0.05) (Fig. 4D). GO enrichment of the Sa-ER RGs revealed significant enrichment in activation of signaling receptor activity, response to external stimulus, regulation of receptor activity, collagen-containing extracellular matrix, extracellular matrix, heparin binding, receptor regulator activity, receptor ligand activity, cytokine activity, and regulation of peptidyl-tyrosine phosphorylation (Fig. 4E). KEGG enrichment of the Sa-ER RGs showed several relevant pathways, such as cytokine–cytokine receptor interaction, viral protein interaction with cytokine and cytokine receptor, pertussis, complement, coagulation cascades, cell adhesion molecules, prion diseases, nicotine addiction, the cAMP signaling pathway, vascular smooth muscle contraction, and amyotrophic lateral sclerosis (Fig. 4F).

**Figure 4:**
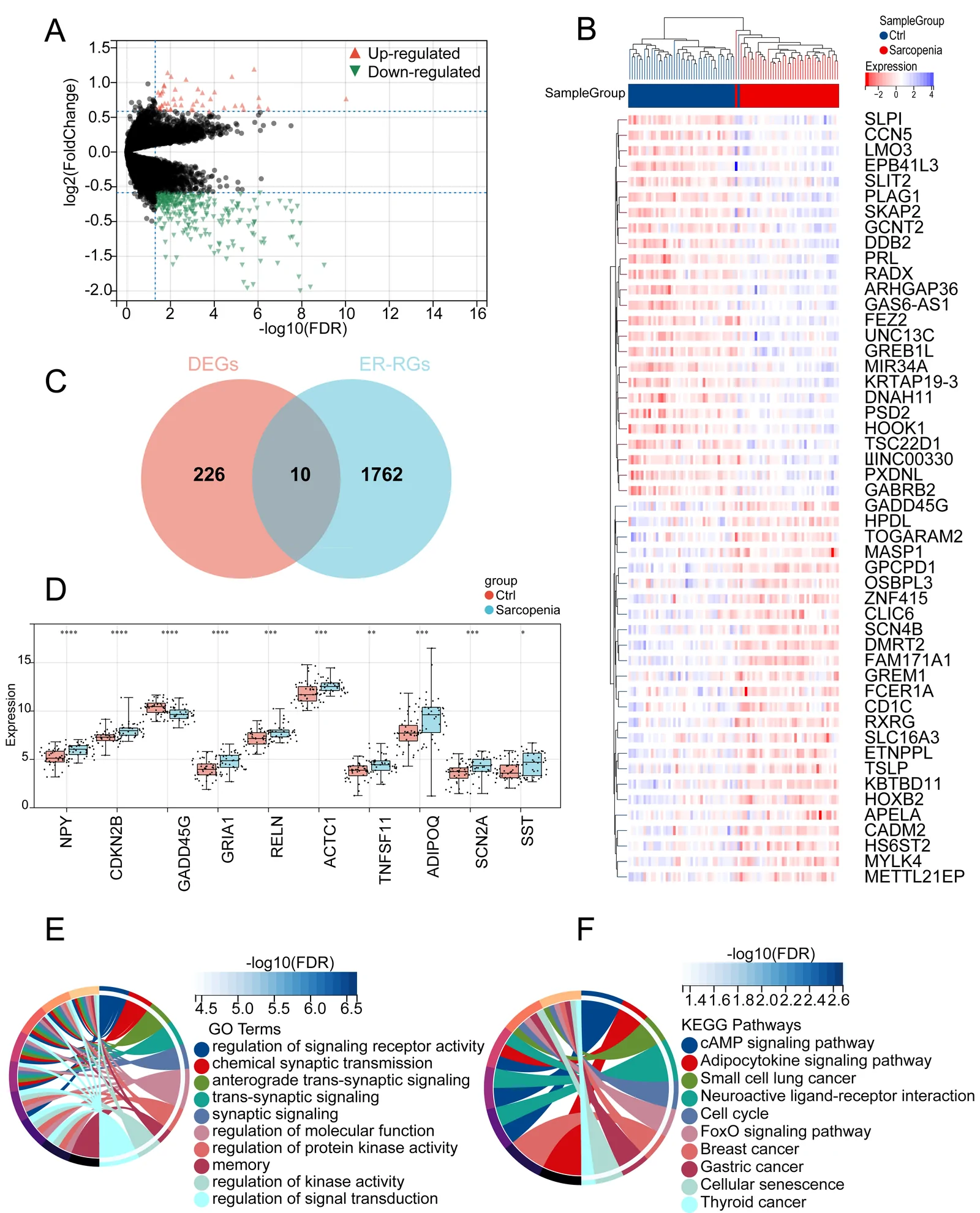
Identification and functional characterization of Sa-ER RGs. (A) Volcano plot of differentially expressed genes ranked by log2 FC and −log10 (FDR). Up-regulated genes are shown in red and down-regulated genes in green. (B) Heatmap of differentially expressed genes across individual samples. Columns represent samples (blue, control; red, sarcopenia) and rows represent genes. (C) Venn diagram showing the overlap between DEGs and ER-RGs. Ten genes were identified as Sa-ER RGs. (D) Box plots comparing expression levels of 10 selected genes between control and sarcopenia groups. (E) GO enrichment of Sa-ER RGs. The outer ring denotes GO terms and the inner ring links to associated genes, color intensity indicates –log10 (*p* value). (F) KEGG enrichment of Sa-ER RGs. Statistical significance is indicated by asterisks (*P < 0.05, **P < 0.01, ***P < 0.001, ****P < 0.0001).

### Machine learning algorithm-based feature hub genes selection

LASSO logistic regression with ten-fold cross-validation was used to optimise the Sa-ER gene cluster, and nine key genes were identified (*λ*.min = 0.023) (Figs. 5A–5B), The Random Forest algorithm identified six feature genes of relative importance by MeanDecreaseGini > 3.5 (Fig. 5C). The 10-fold cross-validation curve in the SVM analysis showed a minimum error rate of 0.11 with 10 features. This indicates that the optimal feature set corresponds to 10 genes (Fig. 5D). The integration of these three algorithms resulted in the identification of five hub genes, namely neuropeptide Y (*NPY*), Cyclin Dependent Kinase Inhibitor 2B (*CDKN2B*), Growth Arrest and DNA Damage-inducible 45 Gamma (*GADD45G*), glutamate ionotropic receptor AMPA type subunit 1 (*GRIA1*), and actin alpha cardiac muscle 1 (*ACTC1*) (Fig. 5E). Chromosomal localization of the hub genes was visualized. Specifically, *GRIA1* localized to chromosome 5, *NPY* to chromosome 7, *CDKN2B* and *GADD45G* both mapped to chromosome 9 in close proximity, and *ACTC1* localized to chromosome 15. The remaining core genes were distributed across other chromosomal regions (Fig. 5F).

**Figure 5:**
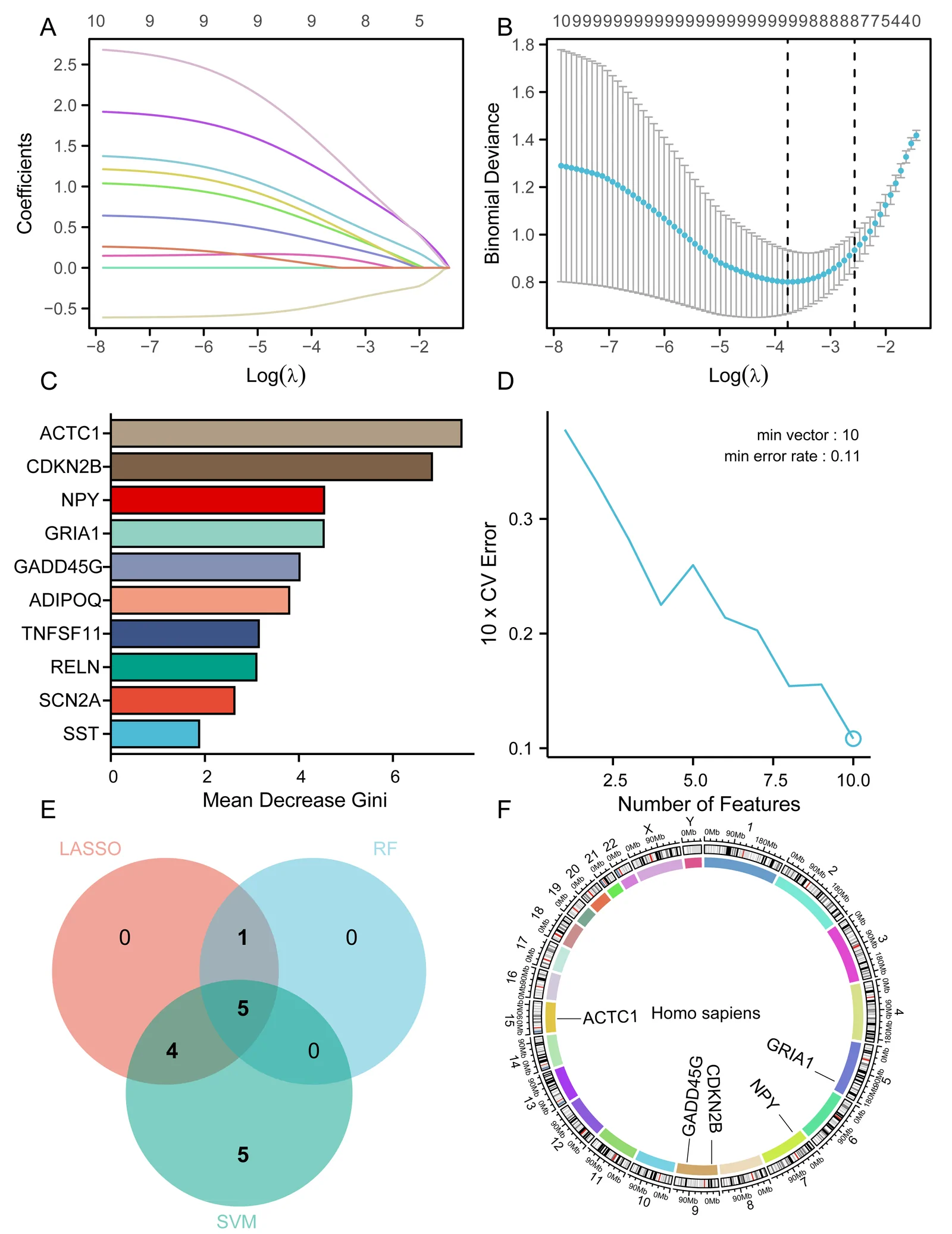
Machine-learning-based selection and chromosomal localization of hub genes. (A) Logistic regression coefficient profiles for LASSO across the log(*λ*) sequence. (B) LASSO regression cross-validation curve. Each red dot shows the average binomial bias, as calculated by cross-validation, at the corresponding log *λ* value. The dashed line on the left represents the optimal lambda value (*λ* min) for the evaluation index. The right-hand dashed line shows the *λ* value of the model (*λ*.1SE), which is within one standard error of the optimal evaluation index value. (C) RF variable importance, ranked by Mean Decrease Gini. (D) Cross-validation error curve for SVM feature selection. The point marked with a blue circle indicates the minimum number of feature variables corresponding to the lowest error rate in the cross-validation results. (E) A Venn diagram showing the overlap of genes retained by LASSO regression, RF and SVM. (F) Chromosome localization map of the hub genes. Each color represents a pair of chromosomes.

### Integrated TF–miRNA-gene, protein–chemical, and gene–gene functional interaction networks of hub genes

Topological network analysis was performed using the CytoNCA plugin, and hub genes were screened according to the criteria of Degree > 2 and BC, CC, and EC values exceeding their respective medians. The TF-miRNA-gene network consisted of 18 nodes and 28 edges. Four transcription factors were predicted to be associated with the hub genes, including TGFB induced factor homeobox 1 (*TGIF1*), interferon regulatory factor 1 (*IRF1*), Serum Response Factor (*SRF*), and POU class 2 homeobox 1 (*POU2F1*), as well as 10 miRNAs (*hsa-miR-25*, *hsa-miR-32*, *hsa-miR-363*, *hsa-miR-367*, *hsa-miR-608*, *hsa-miR-876-3p*, *hsa-miR-92a*, *hsa-miR-92b*, *hsa-miR-140-5p*, *and hsa-miR-495*) (Fig. 6A). Furthermore, the protein-chemical interaction network identified 17 chemicals with reported database associations with the hub genes, including 4-(5-benzo (1,3) dioxol-5-yl-4-pyridin-2-yl-1H-imidazol-2-yl) benzamide, Benzo(a)pyrene, dorsomorphin, Tretinoin, Valproic Acid, Triclosan, Cadmium Chloride, Doxorubicin, Estradiol, sodium arsenite, Aflatoxin B1, Carbamazepine, Sunitinib, Tobacco Smoke Pollution, Phenylmercuric Acetate, Resveratrol, and Diethylhexyl Phthalate (Fig. 6B). In addition, a gene-gene functional interaction network was constructed. Functional annotation indicated enrichment in plasma membrane signaling receptor complex, ionotropic glutamate receptor, complex/neurotransmitter receptor complex, integral component of plasma membrane, and transmembrane transporter complex categories (Fig. 6C).

**Figure 6:**
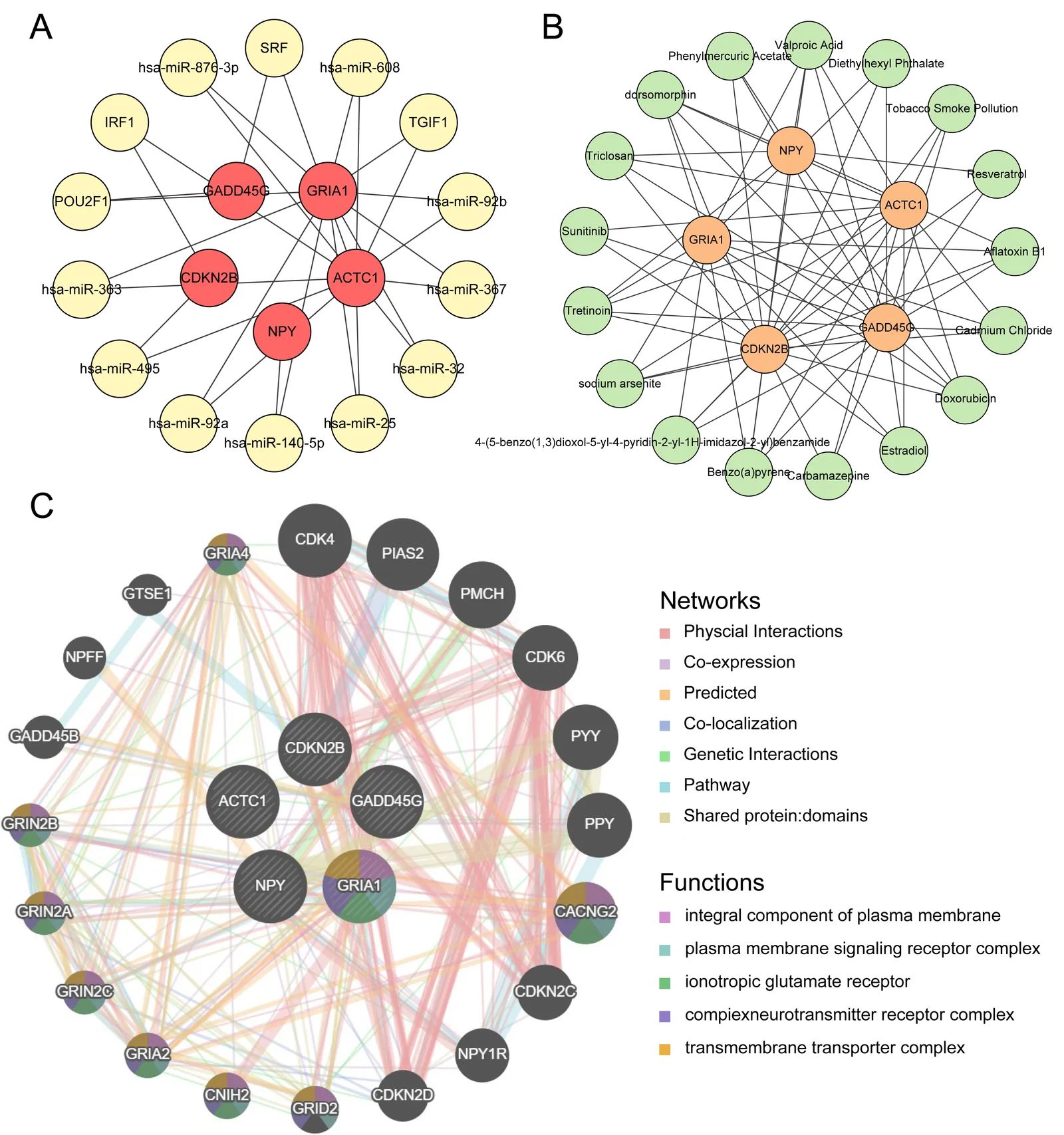
Regulatory networks of the hub genes. (A) TF-miRNA-gene interaction network of hub genes. (B) Protein-chemical interaction network of hub genes. (C) Gene-gene functional interaction network.

### Evaluation of the diagnostic value of hub genes

The diagnostic accuracy of the five hub genes was individually evaluated in the independent GSE1428 cohort. ROC analysis showed that *ACTC1* had an AUC of 0.900 (95% CI [0.704–1.000]), *NPY* had an AUC of 0.725 (95% CI [0.507–0.943]), *GADD45G* had an AUC of 0.733 (95% CI [0.511–0.955]), *GRIA1* had an AUC of 0.625 (95% CI [0.375–0.875]), and *CDKN2B* had an AUC of 0.508 (95% CI [0.251–0.766]). Thus, *ACTC1* showed strong individual discrimination, *NPY* and GADD45G showed moderate discrimination, *GRIA1* showed limited discrimination, and *CDKN2B* showed near-chance individual performance (Fig. S2).

The combined ROC curve, calibration curve, and decision curve analysis provided preliminary evidence regarding discrimination, calibration, and potential net clinical benefit of the multi-gene signature (Figs. 7A–7C). A nomogram was constructed by integrating the scores assigned to each hub gene to quantify the risk score (Fig. 7D).

**Figure 7:**
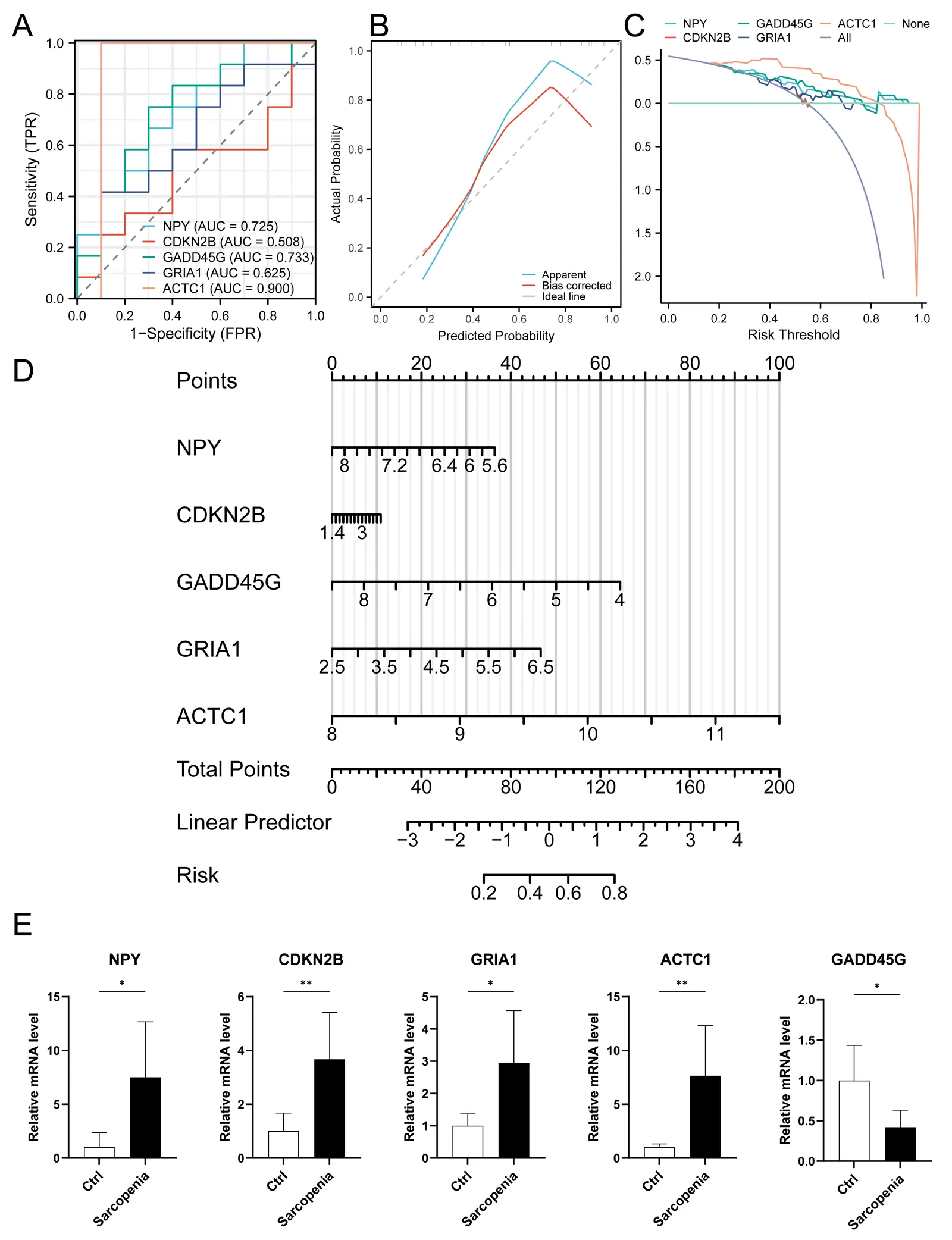
Diagnostic and experimental validation of the 5-gene signature. (A) ROC curve of the combined model. (B) Calibration curve of the combined model. (C) Decision curve analysis of the combined model. (D) Nomogram constructed based on *ACTC1*, *NPY*, *CDKN2B*, *GADD45G*, and *GRIA1*. (E) The mRNA expression levels of the five hub genes. Six independent biological samples were included in each group, and each qRT-PCR reaction was performed in technical triplicate. Data are presented as mean ± SD (*n* = 6). * *p* < 0.05, ** *p* < 0.01.

### Clinical validation of hub gene expression

We next performed qRT-PCR experiments to validate the expression of hub genes in the muscle samples from patients with sarcopenia. The mRNA expression levels of *NPY*, *CDKN2B*, *GRIA1*, and *ACTC1* were significantly upregulated compared with that of the control (*p* < 0.05), whereas *GADD45G* expression was significantly decreased (*p* < 0.05). The qRT-PCR results confirmed the differential expression patterns identified in the integrated transcriptomic datasets (Fig. 7E).

## Discussion

Given its high prevalence and substantial impact on physical function, quality of life, and mortality in older adults, sarcopenia has become a major focus of aging research and biomarker discovery (Gustafsson & Ulfhake, 2024). Although mitochondrial dysfunction, chronic inflammation, impaired proteostasis, autophagy dysregulation, cellular senescence, and neuromuscular degeneration have been implicated in its pathogenesis, clinically applicable molecular biomarkers remain limited (Veronesi et al., 2024). In recent years, disruption of protein quality control has emerged as an important feature of skeletal muscle aging. ER stress and the unfolded protein response (UPR) have been increasingly recognized as key contributors to skeletal muscle atrophy by regulating protein folding, proteasomal degradation, autophagy, inflammation, and mitochondrial homeostasis (Marafon et al., 2022). Under physiological conditions, UPR activation is adaptive and restores ER homeostasis. However, prolonged or excessive ER stress may become maladaptive, promoting muscle catabolism and functional decline (Joshi et al., 2025).

Reticulophagy, also termed ER phagy, is a selective autophagic process that removes damaged or excessive ER fragments and thereby contributes to ER quality control. Compared with ER stress and general autophagy, the role of reticulophagy in human sarcopenia remains poorly defined. Nevertheless, ER phagy is biologically relevant because it directly participates in ER turnover, proteostasis maintenance, and cellular adaptation to stress (Reggiori & Molinari, 2022; Mochida & Nakatogawa, 2022). Autophagy impairment has also been linked to aging phenotypes and skeletal muscle repair capacity (Chen et al., 2022). Therefore, screening sarcopenia-associated molecular signatures within ER stress and reticulophagy related gene networks may provide a rational strategy for biomarker discovery.

In this study, three skeletal muscle transcriptomic datasets were integrated, and 236 DEGs were identified between sarcopenic and control samples. By intersecting these DEGs with ER stress and reticulophagy related genes, ten candidate genes were obtained. Subsequently, three machine learning algorithms identified five hub genes: *ACTC1*, *NPY*, *CDKN2B*, *GADD45G*, and *GRIA1*. Similar multi-step bioinformatics strategies have been used to identify disease-associated diagnostic gene signatures in recent studies. Importantly, these five genes are not canonical ER stress regulators, nor are they classical ER phagy receptors. Therefore, they should be interpreted as biomarkers associated with ER stress- and reticulophagy-related molecular networks rather than as direct mechanistic regulators of ER homeostasis or ER-phagy.

Functional enrichment analyses provided biological context for these hub genes. ECM organization was one of the most prominent enriched processes. Age related skeletal muscle remodeling is accompanied by fibrosis, altered collagen deposition, impaired satellite cell activation, and reduced regenerative capacity. These alterations can compromise force transmission and contribute to progressive muscle dysfunction (Chen et al., 2021; Ge et al., 2025; Li et al., 2025a). The enrichment of ECM related pathways in this study therefore suggests that the identified gene signature may reflect structural remodeling and impaired repair in sarcopenic muscle.

Cytokine-cytokine receptor interaction was also significantly enriched. Chronic low-grade inflammation, or inflammaging, is a key contributor to sarcopenia by promoting proteolysis, anabolic resistance, and impaired regeneration (Dalle, Rossmeislová & Koppo, 2017). Inflammatory signaling also intersects with ER stress pathways through NF-*κ*B, JNK, and related stress-response cascades, suggesting a potential connection between ER homeostasis and inflammatory remodeling in aging muscle (Draganidis et al., 2021; Sharma et al., 2024). Although our data do not directly demonstrate activation of ER stress signaling, the enrichment of inflammatory processes supports the hypothesis that ER-associated molecular changes may be linked to the inflammatory microenvironment of sarcopenic muscle.

Mitochondrial translation and respiratory electron transport pathways were also enriched. Mitochondrial dysfunction is a central hallmark of sarcopenia and contributes to reduced ATP production, excessive reactive oxygen species generation, and impaired muscle regeneration (Marzetti et al., 2024; Huang et al., 2025). The ER and mitochondria communicate through mitochondria-associated membranes, which regulate calcium signaling, energy metabolism, and stress responses. Disruption of ER homeostasis can impair mitochondrial function, while mitochondrial dysfunction can further amplify ER stress, forming a vicious cycle that promotes muscle degeneration (Ji et al., 2025; Li et al., 2025b).

Among the five hub genes, *ACTC1* was significantly upregulated in sarcopenic muscle and validated by qRT-PCR. *ACTC1* encodes α-cardiac actin, a contractile protein that may be re-expressed during muscle injury, remodeling, and regeneration. Its upregulation may therefore reflect compensatory cytoskeletal remodeling in response to chronic structural stress rather than direct ER stress regulation. This interpretation is consistent with studies showing that muscle aging and atrophy involve substantial changes in contractile, cytoskeletal, and regenerative programs (Fernández-Lázaro et al., 2022; Malatesta & Cisterna, 2025).

*NPY* was also significantly upregulated. *NPY* is a neuropeptide involved in energy balance, stress adaptation, appetite regulation, and neuroendocrine signaling. Sarcopenia is increasingly regarded as a systemic disorder involving metabolic, endocrine, neural, and inflammatory interactions rather than an isolated muscle disease (Sousa et al., 2023; Damanti et al., 2025). Thus, elevated *NPY* may reflect altered metabolic and neuroendocrine adaptation in aging muscle. However, direct evidence linking *NPY* to ER stress or reticulophagy in sarcopenia remains lacking, and this association should be interpreted cautiously.

*CDKN2B* may be particularly relevant to aging-related muscle degeneration. *CDKN2B* encodes *p15*^*INK*4*B*^, a cyclin-dependent kinase inhibitor involved in cell-cycle arrest and cellular senescence. Cellular senescence contributes to sarcopenia through impaired regenerative capacity, accumulation of dysfunctional satellite cells, and secretion of senescence-associated inflammatory factors (Xia et al., 2021). Persistent ER stress can also promote senescence-related signaling through stress-response pathways. Therefore, *CDKN2B* upregulation may indicate enhanced senescence activity in sarcopenic muscle and may represent a potential link between cellular stress responses and muscle aging. However, *CDKN2B* showed near-chance individual discrimination in the external validation cohort (AUC = 0.508); therefore, its relevance in the present study should be interpreted primarily as a biological association and as part of the multigene framework rather than as a standalone diagnostic marker.

In contrast, *GADD45G* was significantly downregulated in sarcopenic samples. *GADD45G* participates in DNA damage response, cellular stress adaptation, growth arrest, and apoptosis regulation. Reduced *GADD45G* expression may indicate impaired stress-response capacity in aging skeletal muscle. Since proteostasis failure and reduced cellular resilience are key features of skeletal muscle aging, *GADD45G* downregulation may reflect diminished adaptation to chronic oxidative, metabolic, and proteotoxic stress (Xu & Liu, 2025).

*GRIA1* was another upregulated hub gene. *GRIA1* encodes an AMPA type glutamate receptor subunit mainly involved in excitatory neurotransmission. Increasing evidence suggests that neuromuscular junction degeneration, denervation, and impaired neural-muscle communication are important contributors to sarcopenia (Chen et al., 2024). Therefore, *GRIA1* upregulation may reflect adaptive or maladaptive changes in neuromuscular signaling during skeletal muscle aging, although its relationship with ER homeostasis remains unclear.

The five-gene signature showed heterogeneous individual diagnostic performance. *ACTC1* demonstrated strong discrimination, *NPY* and *GADD45G* showed moderate discrimination, whereas *GRIA1* and particularly *CDKN2B* showed limited individual performance. The multigene framework may nevertheless capture complementary biological information across interconnected processes. The nomogram provides a preliminary framework for individualized prediction, and qRT-PCR validation in clinical muscle biopsies supports the direction of the transcriptomic findings; however, further validation in larger cohorts is required.

Several limitations should be acknowledged. First, this study was mainly based on retrospective public transcriptomic datasets, and the sample size remained limited. The public cohorts were not uniformly defined using contemporary clinical sarcopenia criteria, and aging-related muscle phenotypes may not be fully equivalent to clinically diagnosed sarcopenia. Second, the qRT-PCR validation cohort was small (*n* = 6 per group), and the sarcopenia and control groups differed substantially in age (75.3 ± 11.6 *vs.* 43.7 ± 12.5 years), which represents a potential confounder. In addition, skeletal-muscle transcriptomic profiles may vary according to sarcopenia subtype, comorbidities, and other patient-level driving factors. Third, external validation was performed in GSE1428 on the GPL96 platform, whereas the training datasets were generated on GPL570. Despite platform-specific preprocessing and gene-symbol matching, residual cross-platform effects cannot be excluded. Fourth, the GeneCards Relevance Score > 5 threshold was used as a pragmatic filtering criterion without a formal sensitivity analysis across alternative cutoffs, and this choice may influence the composition of the candidate gene set. Fifth, this study demonstrates associations rather than causality. Whether *ACTC1*, *NPY*, *CDKN2B*, *GADD45G*, and *GRIA1* directly contribute to muscle degeneration or merely represent downstream consequences requires experimental validation. Sixth, none of the five genes are currently recognized as canonical ER stress or ER phagy regulators. Therefore, their mechanistic relationship with ER homeostasis must be tested in cellular and animal models. Finally, transcriptomic changes do not necessarily reflect protein abundance or functional activity, and future studies integrating proteomics, single-cell transcriptomics, spatial transcriptomics, and functional assays are needed.

In conclusion, this study identified and validated five hub genes associated with ER stress and reticulophagy related molecular networks in sarcopenia. Although these genes are not classical ER stress or ER phagy regulators, they may reflect broader ER homeostasis-related alterations during skeletal muscle aging. The five gene signature showed promising diagnostic value and may provide new candidate biomarkers for sarcopenia. These findings offer a foundation for future mechanistic studies investigating the relationship among ER stress, reticulophagy, mitochondrial dysfunction, inflammation, cellular senescence, neuromuscular degeneration, and age-related muscle loss.

## Conclusion

By integrating multiple transcriptomic datasets, machine-learning approaches, and clinical validation, we identified five hub genes associated with ER stress and reticulophagy related molecular networks in sarcopenia. The resulting gene signature demonstrated preliminary diagnostic potential, although the individual contribution of each gene varied substantially in external validation. While the identified genes are not canonical regulators of ER stress or ER phagy, they may serve as indicators of ER homeostasis related molecular disturbances in aging skeletal muscle. These findings expand our understanding of the molecular mechanisms underlying sarcopenia and provide candidate biomarkers for further validation and a basis for future mechanistic investigation.

##  Supplemental Information

10.7717/peerj.21903/supp-1 Supplemental Information 1 Raw data

10.7717/peerj.21903/supp-2 Supplemental Information 2 MIQE checklist

10.7717/peerj.21903/supp-3 Supplemental Information 3 GSEA plot of custom gene set enrichment analysis

10.7717/peerj.21903/supp-4 Supplemental Information 4 Individual ROC, calibration, and decision curve analyses of the five hub genes in the GSE1428 validation cohort

## Additional Information and Declarations

### Data Availability

The following information was supplied regarding data availability: The raw measurements are available in the Supplemental Files. The raw sequence data is available at NCBI: GSE1428

## References

[ref-1] Aragon AA, Tipton KD, Schoenfeld BJ (2023). Age-related muscle anabolic resistance: inevitable or preventable?. Nutrition Reviews.

[ref-2] Bahat G, Ozkok S (2024). The current landscape of pharmacotherapies for sarcopenia. Drugs & Aging.

[ref-3] Bohnert KR, McMillan JD, Kumar A (2018). Emerging roles of ER stress and unfolded protein response pathways in skeletal muscle health and disease. Journal of Cellular Physiology.

[ref-4] Chen H, Boutros PC (2011). VennDiagram: a package for the generation of highly-customizable Venn and Euler diagrams in R. BMC Bioinformatics.

[ref-5] Chen J, Chen H, Dong X, Hui T, Yan M, Ren D, Zou S, Wang S, Fei E, Zhang W, Lai X (2024). Deficiency of skeletal muscle Agrin contributes to the pathogenesis of age-related sarcopenia in mice. Cell Death & Disease.

[ref-6] Chen W, Chen Y, Liu Y, Wang X (2022). Autophagy in muscle regeneration: potential therapies for myopathies. Journal of Cachexia, Sarcopenia and Muscle.

[ref-7] Chen W-J, Lin I-H, Lee C-W, Chen Y-F (2021). Aged skeletal muscle retains the ability to remodel extracellular matrix for degradation of collagen deposition after muscle injury. International Journal of Molecular Sciences.

[ref-8] Chen L-K, Woo J, Assantachai P, Auyeung T-W, Chou M-Y, Iijima K, Jang HC, Kang L, Kim M, Kim S, Kojima T, Kuzuya M, Lee JSW, Lee SY, Lee W-J, Lee Y, Liang C-K, Lim J-Y, Lim WS, Peng L-N, Sugimoto K, Tanaka T, Won CW, Yamada M, Zhang T, Akishita M, Arai H (2020). Asian working group for sarcopenia: 2019 consensus update on sarcopenia diagnosis and treatment. Journal of the American Medical Directors Association.

[ref-9] Chino H, Mizushima N (2023). ER-Phagy: quality and quantity control of the endoplasmic reticulum by autophagy. Cold Spring Harbor Perspectives in Biology.

[ref-10] Coletta G, Phillips SM (2023). An elusive consensus definition of sarcopenia impedes research and clinical treatment: a narrative review. Ageing Research Reviews.

[ref-11] Cruz-Jentoft AJ, Sayer AA (2019). Sarcopenia. The Lancet.

[ref-12] Dalle S, Rossmeislová L, Koppo K (2017). The role of inflammation in age-related sarcopenia. Frontiers in Physiology.

[ref-13] Damanti S, Senini E, De Lorenzo R, Merolla A, Santoro S, Festorazzi C, Messina M, Vitali G, Sciorati C, Manfredi AA, Rovere-Querini P (2025). Molecular constraints of sarcopenia in the ageing muscle. Frontiers in Aging.

[ref-14] Deng B, Liu Y, He P, Wang N, Xu L, Ma J (2026). Shared molecular features and potential diagnostic biomarkers between ulcerative colitis and sarcopenia: an integrative bioinformatics analysis with preliminary experimental validation. Frontiers in Immunology.

[ref-15] Draganidis D, Jamurtas AZ, Chondrogianni N, Mastorakos G, Jung T, Grune T, Papadopoulos C, Papanikolaou K, Papassotiriou I, Papaevgeniou N, Poulios A, Batrakoulis A, Deli CK, Georgakouli K, Chatzinikolaou A, Karagounis LG, Fatouros IG (2021). Low-grade systemic inflammation interferes with anabolic and catabolic characteristics of the aged human skeletal muscle. Oxidative Medicine and Cellular Longevity.

[ref-16] Engebretsen S, Bohlin J (2019). Statistical predictions with glmnet. Clinical Epigenetics.

[ref-17] Fernández-Lázaro D, Garrosa E, Seco-Calvo J, Garrosa M (2022). Potential satellite cell-linked biomarkers in aging skeletal muscle tissue: proteomics and proteogenomics to monitor sarcopenia. Proteomes.

[ref-18] Franz M, Rodriguez H, Lopes C, Zuberi K, Montojo J, Bader GD, Morris Q (2018). GeneMANIA update 2018. Nucleic Acids Research.

[ref-19] Gallot YS, Bohnert KR (2021). Confounding roles of ER stress and the unfolded protein response in skeletal muscle atrophy. International Journal of Molecular Sciences.

[ref-20] Ge X, Jin Y, He J, Jia Z, Liu Y, Xu Y (2025). Extracellular matrix in skeletal muscle injury and atrophy: mechanisms and therapeutic implications. Journal of Orthopaedic Translation.

[ref-21] Giresi PG, Stevenson EJ, Theilhaber J, Koncarevic A, Parkington J, Fielding RA, Kandarian SC (2005). Identification of a molecular signature of sarcopenia. Physiological Genomics.

[ref-22] Gu Z (2022). Complex heatmap visualization. IMeta.

[ref-23] Gu Z, Gu L, Eils R, Schlesner M, Brors B (2014). circlize implements and enhances circular visualization in R. Bioinformatics.

[ref-24] Gustafsson T, Ulfhake B (2024). Aging skeletal muscles: what are the mechanisms of age-related loss of strength and muscle mass, and can we impede its development and progression?. International Journal of Molecular Sciences.

[ref-25] Gustavsson EK, Zhang D, Reynolds RH, Garcia-Ruiz S, Ryten M (2022). ggtranscript: an R package for the visualization and interpretation of transcript isoforms using ggplot2. Bioinformatics.

[ref-26] Hänzelmann S, Castelo R, Guinney J (2013). GSVA: gene set variation analysis for microarray and RNA-seq data. BMC Bioinformatics.

[ref-27] Huang Y, Wang C, Cui H, Sun G, Qi X, Yao X (2025). Mitochondrial dysfunction in age-related sarcopenia: mechanistic insights, diagnostic advances, and therapeutic prospects. Frontiers in Cell and Developmental Biology.

[ref-28] Ji Y, Jiang Q, Chen B, Chen X, Li A, Shen D, Shen Y, Liu H, Qian X, Yao X, Sun H (2025). Endoplasmic reticulum stress and unfolded protein response: roles in skeletal muscle atrophy. Biochemical Pharmacology.

[ref-29] Johnson WE, Li C, Rabinovic A (2007). Adjusting batch effects in microarray expression data using empirical Bayes methods. Biostatistics.

[ref-30] Joshi AS, Tomaz da Silva M, Vuong AT, Xu B, Singh RK, Kumar A (2025). The canonical ER stress IRE1α/XBP1 pathway mediates skeletal muscle wasting during pancreatic cancer cachexia. EMBO Molecular Medicine.

[ref-31] Li N, Chen Y, Wang Q, Liu X, Han C, Qu C, Guan X, Zou W, Wang X, Li A, Liu J, Wang Y (2025a). Microenvironment-driven satellite cell regeneration and repair in aging-related sarcopenia: mechanisms and therapeutic frontiers. Stem Cell Research & Therapy.

[ref-32] Li Y, Ge X, Peng F, Li W, Li JJ (2022). Exaggerated false positives by popular differential expression methods when analyzing human population samples. Genome Biology.

[ref-33] Li X, Zhao X, Qin Z, Li J, Sun B, Liu L (2025b). Regulation of calcium homeostasis in endoplasmic reticulum-mitochondria crosstalk: implications for skeletal muscle atrophy. Cell Communication and Signaling.

[ref-34] Liu D, Sartor MA, Nader GA, Pistilli EE, Tanton L, Lilly C, Gutmann L, IglayReger HB, Visich PS, Hoffman EP, Gordon PM (2013). Microarray analysis reveals novel features of the muscle aging process in men and women. The Journals of Gerontology: Series A.

[ref-35] Malatesta M, Cisterna B (2025). Aging in the skeletal muscle revealed by molecular immunohistochemical imaging. International Journal of Molecular Sciences.

[ref-36] Mandrekar JN (2010). Receiver operating characteristic curve in diagnostic test assessment. Journal of Thoracic Oncology.

[ref-37] Marafon BB, Pinto AP, Ropelle ER, De Moura LP, Cintra DE, Pauli JR, Da Silva ASR (2022). Muscle endoplasmic reticulum stress in exercise. Acta Physiologica.

[ref-38] Marmondi F, Ferrando V, Filipas L, Codella R, Ruggeri P, Torre ALa, Faelli EL, Bonato M (2025). Exercise-induced biomarker modulation in sarcopenia: from inflamm-aging to muscle regeneration. Sports.

[ref-39] Marzetti E, Calvani R, Coelho-Júnior HJ, Landi F, Picca A (2024). Mitochondrial quantity and quality in age-related sarcopenia. International Journal of Molecular Sciences.

[ref-40] Mochida K, Nakatogawa H (2022). ER-phagy: selective autophagy of the endoplasmic reticulum. The EMBO Reports.

[ref-41] Ooi H, Welch C (2024). Obstacles to the early diagnosis and management of sarcopenia: current perspectives. Clinical Interventions in Aging Volume.

[ref-42] Paez HG, Pitzer CR, Alway SE (2023). Age-related dysfunction in proteostasis and cellular quality control in the development of sarcopenia. Cell.

[ref-43] Petermann-Rocha F, Balntzi V, Gray SR, Lara J, Ho FK, Pell JP, Celis-Morales C (2022). Global prevalence of sarcopenia and severe sarcopenia: a systematic review and meta-analysis. Journal of Cachexia, Sarcopenia and Muscle.

[ref-44] Ran S, Li Z, Lin X, Liu B (2025). Identifying semaphorin 3C as a biomarker for sarcopenia and coronary artery disease *via* bioinformatics and machine learning. Archives of Gerontology and Geriatrics.

[ref-45] Raue U, Trappe TA, Estrem ST, Qian H-R, Helvering LM, Smith RC, Trappe S (2012). Transcriptome signature of resistance exercise adaptations: mixed muscle and fiber type specific profiles in young and old adults. Journal of Applied Physiology.

[ref-46] Reggiori F, Molinari M (2022). ER-phagy: mechanisms, regulation, and diseases connected to the lysosomal clearance of the endoplasmic reticulum. Physiological Reviews.

[ref-47] Ritchie ME, Phipson B, Wu D, Hu Y, Law CW, Shi W, Smyth GK (2015). limma powers differential expression analyses for RNA-sequencing and microarray studies. Nucleic Acids Research.

[ref-48] Robin X, Turck N, Hainard A, Tiberti N, Lisacek F, Sanchez J-C, Müller M (2011). pROC: an open-source package for R and S+ to analyze and compare ROC curves. BMC Bioinformatics.

[ref-49] Sharma AR, Chatterjee S, Lee Y-H, Lee S-S (2024). Targeting crosstalk of signaling pathways among muscles-bone-adipose tissue: a promising therapeutic approach for sarcopenia. Aging and Disease.

[ref-50] Shur NF, Creedon L, Skirrow S, Atherton PJ, MacDonald IA, Lund J, Greenhaff PL (2021). Age-related changes in muscle architecture and metabolism in humans: the likely contribution of physical inactivity to age-related functional decline. Ageing Research Reviews.

[ref-51] Sousa D, Lopes E, Rosendo-Silva D, Matafome P (2023). The bidirectional relationship of NPY and mitochondria in energy balance regulation. Biomedicines.

[ref-52] Subramanian A, Tamayo P, Mootha VK, Mukherjee S, Ebert BL, Gillette MA, Paulovich A, Pomeroy SL, Golub TR, Lander ES, Mesirov JP (2005). Gene set enrichment analysis: a knowledge-based approach for interpreting genome-wide expression profiles. Proceedings of the National Academy of Sciences of the United States of America.

[ref-53] Taminau J, Meganck S, Lazar C, Steenhoff D, Coletta A, Molter C, Duque R, Schaetzen V de, Weiss Solís DY, Bersini H, Nowé A (2012). Unlocking the potential of publicly available microarray data using inSilicoDb and inSilicoMerging R/Bioconductor packages. BMC Bioinformatics.

[ref-54] Tang H, Inoki K, Brooks SV, Okazawa H, Lee M, Wang J, Kim M, Kennedy CL, Macpherson PCD, Ji X, Van Roekel S, Fraga DA, Wang K, Zhu J, Wang Y, Sharp ZD, Miller RA, Goldman DT, Guan K-L, Shrager BJ (2019). mTORC1 underlies age-related muscle fiber damage and loss by inducing oxidative stress and catabolism. Aging Cell.

[ref-55] Tang Y, Li M, Wang J, Pan Y, Wu F-X (2015). CytoNCA: a cytoscape plugin for centrality analysis and evaluation of protein interaction networks. Biosystems.

[ref-56] Veronesi F, Salamanna F, Borsari V, Ruffilli A, Faldini C, Giavaresi G (2024). Unlocking diagnosis of sarcopenia: the role of circulating biomarkers—a clinical systematic review. Mechanisms of Ageing and Development.

[ref-57] Wang D, Yang Y, Zou X, Zhang J, Zheng Z, Wang Z (2020). Antioxidant apigenin relieves age-related muscle atrophy by inhibiting oxidative stress and hyperactive mitophagy and apoptosis in skeletal muscle of mice. The Journals of Gerontology: Series A.

[ref-58] Wang Y, Zhuang H, Jiang X, Zou R, Wang H, Fan Z (2023). Unveiling the key genes, environmental toxins, and drug exposures in modulating the severity of ulcerative colitis: a comprehensive analysis. Frontiers in Immunology.

[ref-59] Wilson A, McCormick C (2025). Reticulophagy and viral infection. Autophagy.

[ref-60] Wu T, Hu E, Xu S, Chen M, Guo P, Dai Z, Feng T, Zhou L, Tang W, Zhan L, Fu X, Liu S, Bo X, Yu G (2021). clusterProfiler 4.0: a universal enrichment tool for interpreting omics data. The Innovation.

[ref-61] Xia Y, Liu Y, Yang C, Simeone DM, Sun T-T, De Graff DJ, Tang M, Zhang Y, Wu X-R (2021). Dominant role of CDKN2B/p15INK4B of 9p21.3 tumor suppressor hub in inhibition of cell-cycle and glycolysis. Nature Communications.

[ref-62] Xu M, Liu X (2025). Cisplatin-induced skeletal muscle atrophy: biomolecular mechanisms and the protective role of exercise-induced myokines. Biomolecules.

[ref-63] Zhang Y, Pan X, Sun Y, Geng Y, Yu X-Y, Li Y (2018). The molecular mechanisms and prevention principles of muscle atrophy in aging. Advances in Experimental Medicine and Biology.

