# Supplementary material for: Identification of Endoplasmic reticulum Homeostasis-associated diagnostic biomarkers for Sarcopenia through integrated Bioinformatics and Experimental Validation
Source: PeerJ. 2026 Nov 2;14:e21903. doi: 10.7717/peerj.21903 (PMC13738190; doi:10.7717/peerj.21903)

Supplementary Figure S1

GSEA plot of custom gene set enrichment analysis

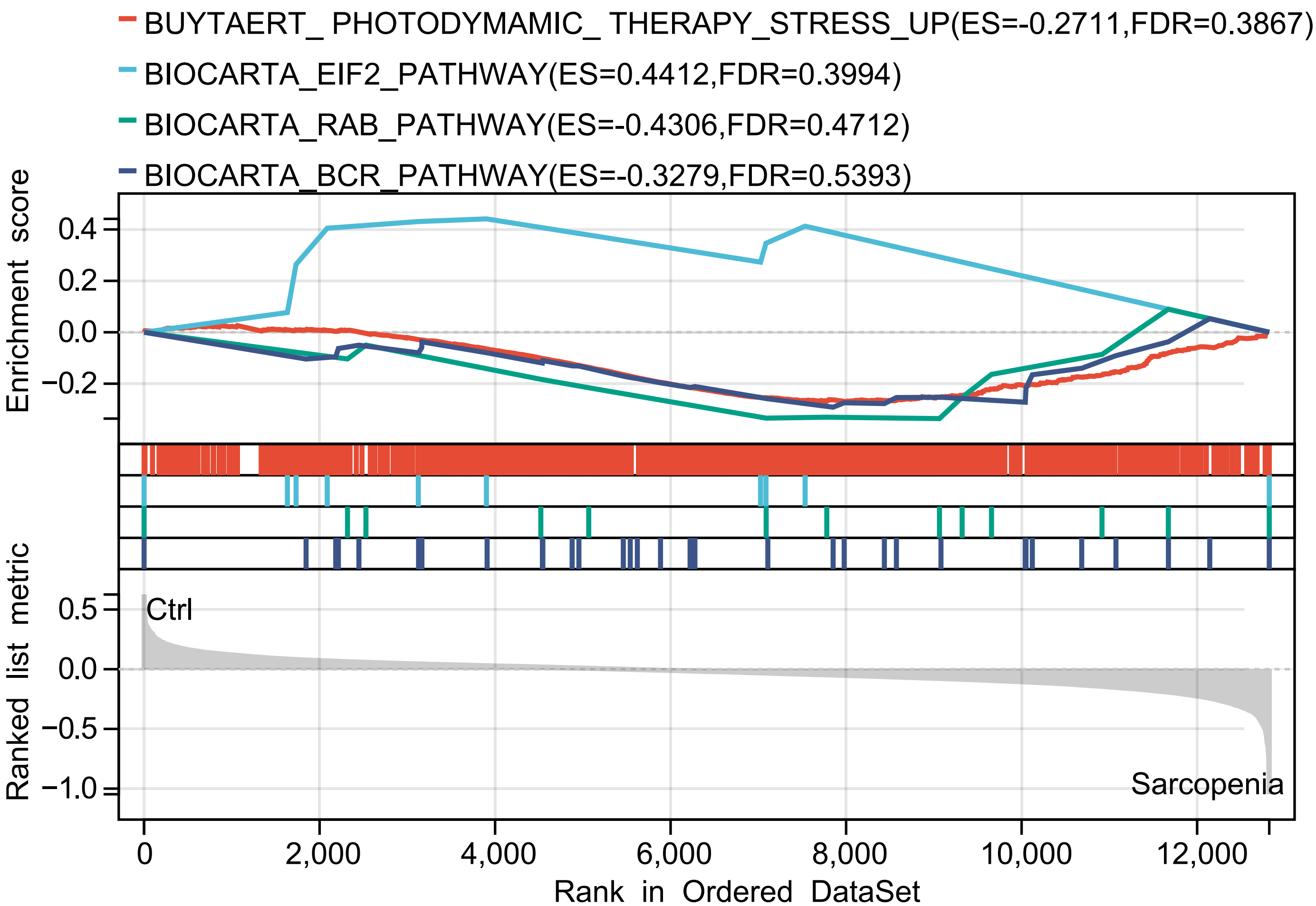

Supplement: Supplementary file 1 [file peerj-14-21903-s003.pdf]
