# Supplementary material for: Identification of Endoplasmic reticulum Homeostasis-associated diagnostic biomarkers for Sarcopenia through integrated Bioinformatics and Experimental Validation
Source: PeerJ. 2026 Nov 2;14:e21903. doi: 10.7717/peerj.21903 (PMC13738190; doi:10.7717/peerj.21903)
Supplement: Supplementary file 2 [file peerj-14-21903-s004.pdf]

Supplementary Figure S2

Individual ROC, calibration, and decision curve analyses of the five hub genes in the GSE1428 validation cohort

(A1–E1) Receiver operating characteristic (ROC) curves for *ACTC1*, *NPY*, *CDKN2B*, *GADD45G*, and *GRIA1*, respectively. The corresponding area under the curve (AUC) values and 95% confidence intervals are shown in each panel. (A2–E2) Calibration curves for the corresponding hub genes, showing the agreement between predicted and observed probabilities. (A3–E3) Decision curve analyses (DCA) for the corresponding hub genes, illustrating the net benefit across a range of threshold probabilities.

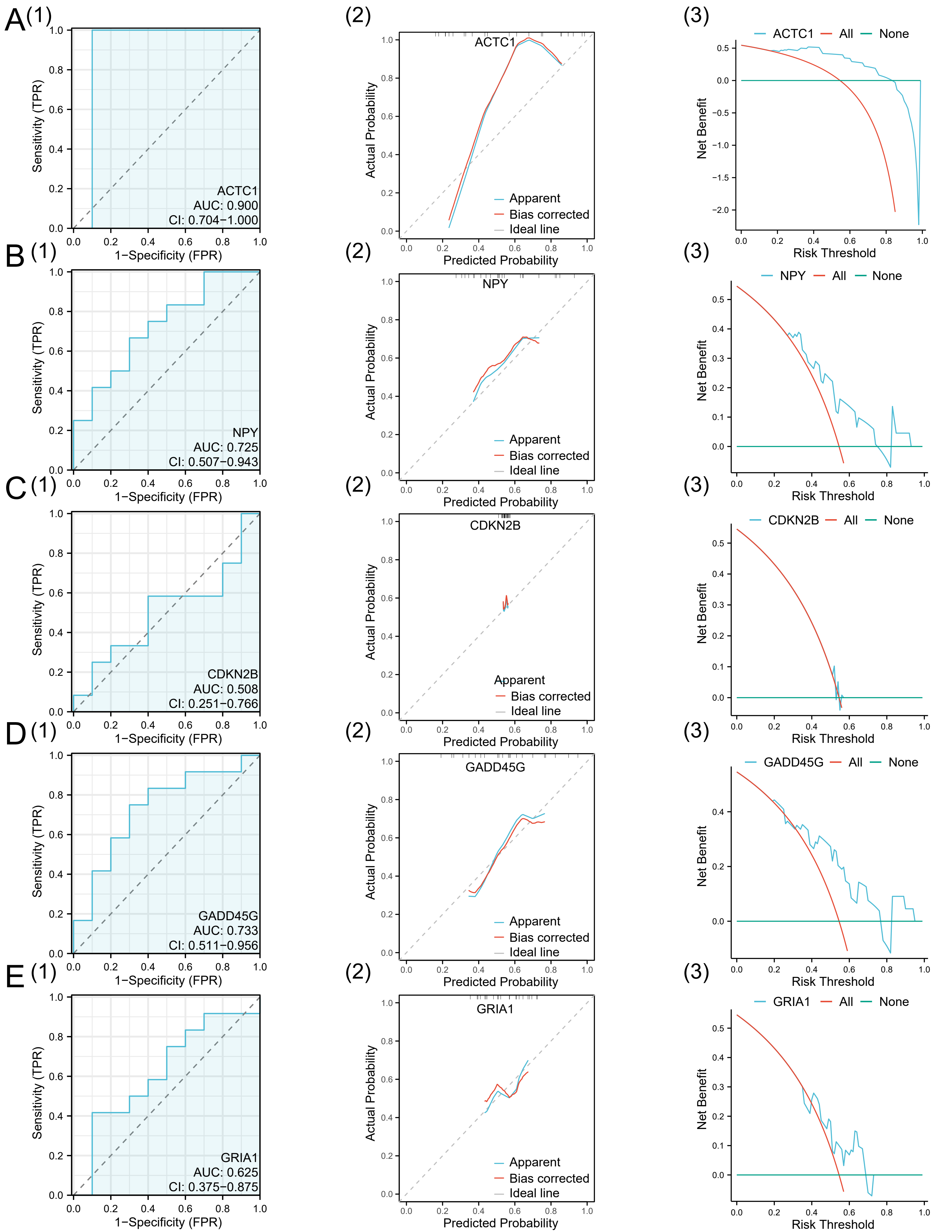
A (1)
ACTC1
AUC: 0.900
CI: 0.704−1.000
Sensitivity (TPR)
1−Specificity (FPR)
(2)
ACTC1
Actual Probability
Predicted Probability
Apparent
Bias corrected
Ideal line
(3)
ACTC1 All None
Net Benefit
Risk Threshold
B (1)
NPY
AUC: 0.725
CI: 0.507−0.943
NPY
CDKN2B
AUC: 0.508
CI: 0.251−0.766
C
D
GADD45G
AUC: 0.733
CI: 0.511−0.956
E
GRIA1
AUC: 0.625
CI: 0.375−0.875
